# S3‐Leitlinie zur Therapie der Psoriasis vulgaris, adaptiert von EuroGuiDerm – Teil 1: Therapieempfehlungen und Monitoring

**DOI:** 10.1111/ddg.16002_g

**Published:** 2026-01-14

**Authors:** Alexander Nast, Andreas Altenburg, Matthias Augustin, Frank Bachmann, Wolf‐Henning Boehncke, Markus Cornberg, Hilte Geerdes‐Fenge, Brit Häcker, Peter Härle, Joachim Klaus, Michaela Köhm, Arno Köllner, Ulrich Mrowietz, Hans‐Michael Ockenfels, Antonia Pennitz, Sandra Philipp, Thomas Richter, Thomas Rosenbach, Tom Schaberg, Martin Schlaeger, Gerhard Schmid‐Ott, Michael Sebastian, Karisa Thölken, Ralph von Kiedrowski, Uwe Willuhn, Christoph Zeyen

**Affiliations:** ^1^ Division of Evidence‐Based Medicine (dEBM) Klinik für Dermatologie Venerologie und Allergologie Charité ‐ Universitätsmedizin Berlin corporate member of Freie Universität Berlin and Humboldt‐Universität zu Berlin Berlin Deutschland; ^2^ Hochschulklinik für Dermatologie Venerologie und Allergologie Städtisches Klinikum Dessau Dessau Deutschland; ^3^ Institut für Versorgungsforschung in der Dermatologie und bei Pflegeberufen (IVDP) Universitätsklinikum Hamburg‐Eppendorf (UKE) Hamburg Deutschland; ^4^ Hautzentrum Berlin Siddi & Bachmann Berlin Deutschland; ^5^ Service de Dermatologie et Vénéréologie Hôpitaux Universitaires de Genève Genf Schweiz; ^6^ Klinik für Gastroenterologie Hepatologie und Endokrinologie an der Medizinischen Hochschule Hannover Hannover Deutschland; ^7^ Klinik für Infektiologie und Tropenmedizin Department für Innere Medizin Universitätsmedizin Rostock Rostock Deutschland; ^8^ Deutsches Zentralkomitee zur Bekämpfung der Tuberkulose (DZK) Berlin Deutschland; ^9^ Rheumatologisches Zentrum nach G‐BA Klinik für Rheumatologie Klinische Immunologie und Physikalische Therapie Marienhaus Klinikum Mainz Mainz Deutschland; ^10^ Deutscher Psoriasis Bund Hamburg Deutschland; ^11^ Abteilung Rheumatologie Immunologie ‐ Entzündungsmedizin Universitätsklinikum Frankfurt Goethe‐Universität Frankfurt am Main Deutschland; ^12^ Fraunhofer Institut für Translationale Medizin und Pharmakologie ITMP Frankfurt Frankfurt am Main Deutschland; ^13^ Hautarztpraxis Duisburg Deutschland; ^14^ Psoriasis‐Zentrum Klinik für Dermatologie Venerologie Allergologie Universitätsklinikum Schleswig‐Holstein Campus Kiel Kiel Deutschland; ^15^ Haut‐ und Allergieklinik Klinikum Hanau Hanau Deutschland; ^16^ Hautarztpraxis Oranienburg Deutschland; ^17^ Helios Versorgungszentren GmbH MVZ Gotha Gotha Deutschland; ^18^ Hautarztpraxis Osnabrück Deutschland; ^19^ Hautarztpraxis Oldenburg Deutschland; ^20^ Berolina Klinik Löhne Deutschland; ^21^ Hautarztpraxis Mahlow Deutschland; ^22^ Universitätsklinik für Dermatologie Universitätsklinikum Augsburg Augsburg Deutschland; ^23^ Hautarztpraxis Selters Deutschland

**Keywords:** Empfehlungen, Monitoring, Psoriasis, Scores, Ziele, Monitoring, psoriasis, recommendations, scores, targets

## Abstract

Der vorliegende Teil 1 der Aktualisierung der S3‐Leitlinie zur Therapie der Psoriasis vulgaris umfasst die Abschnitte „Therapieempfehlungen“, „Therapieziele“ sowie die „Monitoringempfehlungen“ zu den Therapien. Die Grundlage bildet die aktuelle Cochrane‐Netzwerkmetaanalyse, deren Ergebnisse ebenfalls dargestellt werden.

Es wird empfohlen, Wirksamkeit, Sicherheit, Komorbidität und individuelle Patientenfaktoren bei der Auswahl einer systemischen Therapie bei Psoriasis vulgaris zu berücksichtigen. Der Entscheidungskorridor wird in der Übersicht der Therapieoptionen dargestellt und rückt in der aktuellen Fassung die Möglichkeit einer Erstlinientherapie mit Biologika beziehungsweise neuen zielgerichteten kleinen Molekülen weiter nach vorne.

Die Leitlinie betont die Bedeutung standardisierter Instrumente zur Bestimmung des Schweregrades sowie patientenzentrierter Therapieziele. Als Mindestziel gilt ein *Psoriasis‐Area‐and‐Severity‐Index* (PASI) 75‐Ansprechen, während PASI 90 oder ein absoluter PASI <2 als anzustrebende Therapieziele gelten. Aufgenommen wurden die seit der letzten Fassung neu zugelassenen Wirkstoffe Bimekizumab und Deucravacitinib, für die spezifische Anwendungshinweise ergänzt wurden. Unter den bestehenden Kapiteln wurden insbesondere die Anwendungshinweise zu Methotrexat (MTX) umfangreich überarbeitet.

## HINWEISE ZUR ANWENDUNG DER LEITLINIE

Diese Publikation beinhaltet ausgewählte Textpassagen, in denen besonders relevante Änderungen vorgenommen wurden. Neben diesen hier in Teil 1 dargestellten Abschnitten, enthält Teil 2 zudem die „Hilfestellungen für besondere klinische Situationen und bei Vorliegen von Komorbiditäten“

Die Langfassung der Leitlinie befindet sich auf den Seiten der AWMF (https://register.awmf.org/de/leitlinien/detail/013‐001). Insbesondere die Informationen des Kapitels „Hinweise zur Anwendung der Leitlinie/Haftungsausschluss“ der Langfassung sind für die Anwendung der in dieser Kurzfassung dargestellten Leitlinienempfehlungen zu beachten. Auf den Seiten der AWMF befinden sich auch folgende Begleitdokumente zur Version 8 der Leitlinie: Appendix A („Empfehlungen zu topischer Therapie“, „Phototherapie“, „sonstige Therapien“, „Schnittstellendefinition“), Evidenzbericht, Leitlinienreport mit Angaben zu Interessenkonflikten, PowerPoint Foliensatz zur Leitlinienimplementierung.

## WAS IST NEU?

Die Abschnitte Schweregrad und Therapieziele, allgemeine Empfehlungen zur Einleitung und Auswahl einer systemischen Therapie, Übersicht der Therapieoptionen, sowie der Abschnitt zur Darstellung der Wirksamkeit und Sicherheit der Therapien wurde aufbauend auf der Cochrane Netzwerkmetaanalyse überabeitet. Bimekizumab und Deucravacitinib wurden in die Leitlinie aufgenommen und mit entsprechenden Hinweisen zur Durchführung der Therapie versehen. Das Kapitel zu Methotrexat wurde inhaltlich überarbeitet. In dem Abschnitt „Hilfestellungen für besondere klinische Situationen und bei Vorliegen von Komorbiditäten“ wurden die Kapitel Screening auf Tuberkulose, Management der Psoriasis bei Patienten mit latenter Tuberkulose beziehungsweise mit Virushepatitis umfangreich überarbeitet. Die Empfehlungen zur „Systemtherapie bei Patienten mit malignen Erkrankungen“ wurden ebenfalls verändert. Neu ergänzte Textpassagen und wesentliche inhaltliche Änderungen sind in der Langfassung der Leitlinie durch blaue Textfarbe markiert. Textpassagen, die entfallen sind, wurden zur Gewährung der Übersichtlichkeit nicht weiter hervorgehoben.

*Für die Abschnitte „Hinweise zur Anwendung der Leitlinie/Haftungsausschluss“ (diese gelten in gleichem Maße für die vorliegenden Kurzfassungen), „Finanzierung“, „Gegenstand und Ziele der Leitlinie“, „Patientenzielgruppe und zu behandelnde Fragestellungen“ sowie „Zielgruppe dieser Leitlinie“ siehe Langfassung*.

## SCHWEREGRAD UND THERAPIEZIELE

### Bestimmung der Krankheitsaktivität

Trotz einiger Nachteile ist der *Psoriasis Area and Severity Index* (PASI), der erstmals 1978 als Endpunkt in einer Retinoidstudie eingeführt wurde, das am besten etablierte Instrument zur Erfassung der Schwere von Hautsymptomen bei Patienten mit Psoriasis.^1^ Ein weiteres Instrument, das in der täglichen Versorgung eine rasche Bestimmung des Schweregrades einer Psoriasis vulgaris ermöglicht, ist die standardisierte globale Einschätzung des Arztes (*Physician Global Assessment*; PGA). Es existieren verschiedene PGA mit unterschiedlichen Begrifflichkeiten und Skalen. Eine weitere Möglichkeit zur Erfassung der Krankheitsschwere ist die *Body Surface Area* (BSA), eine Schätzung des Prozentsatzes der erkrankten Körperoberfläche.^2^ Daneben stellt die gesundheitsbezogene Lebensqualität (*health‐related quality of life*, HRQoL) eine wichtige Zielgröße dar, die sowohl zur Beurteilung der Krankheitsschwere als auch als Endpunkt in klinischen Studien verwendet wird. Um die Auswirkungen einer Psoriasis vulgaris auf die HRQoL zu erfassen, wurde bisher überwiegend der *Dermatology Life Quality Index* (DLQI) eingesetzt. Die Konstruktvalidität des DLQI wird jedoch in Frage gestellt: Items, die mit der Option „Frage betrifft mich nicht“ beantwortet werden, werden so bewertet, als hätten sie keine Auswirkungen auf das Leben der Patienten und tragen deshalb keine Punkte zum Gesamtscore bei. Dies führt dazu, dass der Gesamtscore niedriger ausfällt als in bestimmten Fällen zu rechtfertigen ist.^3^ Ergänzend zum DLQI steht der *WHO‐5* zur Verfügung, der das Konzept der menschenzentrierten Gesundheitsversorgung abbildet.^4^ Mit dem *ActiPso* steht ein Instrument zur Verfügung, das die Krankheitsaktivität der Psoriasis abbildet.^5^

### Definition des Schweregrades

Die Bestimmung des Schweregrades bei der Psoriasis ist ein komplexes Unterfangen, bei dem viele klinische aber auch die gesundheitsbezogene Lebensqualität betreffende Aspekte berücksichtigt werden müssen. Die derzeit verfügbaren Instrumente weisen verschiedene Unzulänglichkeiten auf und erfassen – wie auch von Patienten häufig berichtet – das Gesamtbild der Erkrankung in seiner Komplexität nicht vollständig. Wie von Mara Maccarone und Ray Jobling, Patientenvertreter der *European Dermatology Forum* (EDF) *Guidelines* 2015, treffend formuliert wurde: „*Severity has become defined technically and bureaucratically, in terms of scores derived from instruments like PASI, DLQI, and Skindex‐25. These simply fail to capture the seriousness of psoriasis as experienced by those who have the disease*.“ Ins Deutsche übersetzt: „Die Schweregrade wurden *technisch und bürokratisch* definiert, basierend auf Bewertungen durch Instrumente wie beispielsweise PASI, DLQI und Skindex‐29. Diese erfassen jedoch nicht angemessen die Ernsthaftigkeit von Psoriasis, wie sie von denjenigen erlebt wird, die an der Krankheit leiden.“

Die aktuell gebräuchlichste Definition des Schweregrades der Psoriasis ist stark beeinflusst durch die in klinischen Studien übliche Einteilung und wurde zuletzt in einem Europäischen Konsensusprojekt im Jahre 2011 umfangreich diskutiert und konsentiert.

**Table:** 

ii.‐1 | modifiziert [2025] **Definition des Schweregrades der Psoriasis** Leichte Psoriasis:^6^ BSA ≤ 10 und PASI ≤10 und DLQI ≤10 Mittelschwere bis schwere Psoriasis:^6^ (BSA > 10 oder PASI > 10) und DLQI > 10 Beim Vorliegen folgender Kriterien erfolgt zudem ebenfalls eine Einordnung als mittelschwere bis schwere Psoriasis (*Upgrade*‐Kriterien):^6^ Ausgeprägte Erkrankung von sichtbaren Arealen, ausgeprägte Erkrankung der Kopfhaut, Erkrankung des Genitalbereichs, Erkrankung der Handflächen und Fußsohlen, Onycholyse oder Onychodystrophie von mindestens zwei Fingernägeln, Jucken und damit einhergehendes Kratzen, therapieresistente Plaques.^6^ Eine besondere Schwere der Psoriasis besteht unter anderem bei PASI ≥ 20, DLQI ≥ 15; rascher Befundverschlechterung, schwerer Beteiligung der Hände und/oder Füße, der Kopfhaut, des Gesichts, der Nägel oder des Genitalbereichs.	**Statement**	starker konsens Konsensbasiert

### Therapieziele

Das grundsätzliche Ziel jeder Therapie ist die Erscheinungsfreiheit, das heißt die Abwesenheit von kutanen Symptomen der Psoriasis. Jedoch kann dieses Ziel derzeit realistischerweise nicht bei allen Patienten erreicht werden.

Für die erfolgreiche Etablierung von Therapiezielen ist es wichtig, ein Mindestziel zu definieren, dass bei einer Behandlung erreicht werden muss. Wird diese „niedrigste Hürde“ nach einem festgelegten Zeitpunkt nicht erreicht, muss die Therapie angepasst werden. Eine Anpassung kann beispielsweise durch Dosissteigerung, Einleitung einer Kombinationstherapie oder auch durch das Umsetzen auf ein anderes Medikament oder Verfahren erreicht werden. Therapieziele sind individuell mit den Patienten in gemeinsamer Entscheidungsfindung festzulegen.

**Table:** 

iii.‐1 | geprüft [2025] Eine PASI 75‐Antwort ist das minimale Therapieziel am Ende der Induktionstherapie der Psoriasis, das im weiteren Verlauf der Behandlung in regelmäßigen Abstanden weiter überprüft werden sollte.	**Statement**	starker konsens ^*^ Konsensbasiert
iii.‐2 | neu [2025] Als anzustrebendes Therapieziel soll ein PASI 90 beziehungsweise ein absoluter PASI <2 oder DLQI ≤ 1 erreicht werden.	**↑↑**	starker konsens ^*^ Konsensbasiert
iii.‐3 | geprüft [2025] Bei Vorliegen von Kriterien wie einer ausgeprägten Erkrankung von sichtbaren Arealen, ausgeprägter Erkrankung der Kopfhaut, Erkrankung des Genitalbereichs, Erkrankung der Handflächen und Fußsohlen, Onycholyse oder Onychodystrophie von mindestens zwei Fingernägeln, Jucken und damit einhergehendem Kratzen, oder dem Vorliegen therapieresistenter Plaques wird empfohlen, das Erreichen des individuell für diese Form der Beteiligung festgelegte Therapieziel (zum Beispiel unter Verwendung entsprechender Scores wie dem *Nail Psoriasis Severity Index* [NAPSI]) zu prüfen und die Therapie bei Nichterreichen entsprechend anzupassen.	**Statement**	starker konsens ^*^ Konsensbasiert

* Fünf Enthaltungen aufgrund von Interessenkonflikten.

Für schnell wirkende Medikamente sollte die Überprüfung des Erreichens von Therapiezielen am Ende der Induktionstherapie nach 10 bis 12 Wochen, bei Medikamenten mit langsam einsetzender Wirkung nach 16 bis 24 Wochen erfolgen. Das genannte Zeitfenster erfasst dabei nicht immer die maximale, klinische Wirksamkeit. Während der Erhaltungstherapie findet die Überprüfung in den auch für das Therapiemonitoring empfohlenen Intervallen, in der Regel alle 8 bis 12 Wochen, statt.

Einen chronischen Verlauf der Psoriasis zu vermeiden, stellt ein weiteres Therapieziel dar, welches an Aufmerksamkeit gewinnt. Aktuell laufen Studien, um einen möglichen Einfluss durch eine besonders frühe Therapie auf die Chronizität der Erkrankung zu untersuchen.

Abschnitt „Zeit bis zum Wirkungseintritt“: siehe Langfassung.

## METHODIK

*Für weitere Informationen, siehe auch Leitlinienreport* (https://register.awmf.org/de/leitlinien/detail/013‐001).

Standardisierte Begriffe adaptiert von der *GRADE Working Group* wurden zur einheitlichen Formulierung aller Empfehlungen der neu überarbeiteten Abschnitte verwendet (Tabelle 1).^7^

**TABELLE 1:** standardisierte Empfehlungsformulierungen (adaptiert nach^8^, ^9^, ^10^).

Stärke	Formulierung	Symbole	Implikationen
**Starke** Empfehlung für eine Vorgehensweise	„wird empfohlen“ / „wir empfehlen“	↑↑	Wir sind der Auffassung, dass alle oder fast alle informierten Menschen eine Entscheidung zugunsten dieser Intervention treffen würden. Kliniker müssen sich weniger Zeit für den Prozess der Entscheidungsfindung mit den Patienten nehmen und können diese Zeit stattdessen für die Überwindung von Barrieren bei der Implementierung und der Therapieadhärenz einsetzen. In den meisten klinischen Situationen kann die Empfehlung als allgemeine Vorgehensweise übernommen werden.
**Schwache** Empfehlung für eine Vorgehensweise	„kann empfohlen werden“	↑	Wir sind der Auffassung, dass die meisten informierten Menschen, ein substanzieller Anteil jedoch nicht, eine Entscheidung zugunsten dieser Intervention treffen würden. Kliniker und andere Anbieter von Gesundheitsleistungen müssen sich mehr Zeit für den Prozess der Entscheidungsfindung mit den Patienten nehmen. Entscheidungsprozesse im Gesundheitssystem erfordern eine tiefgehende Diskussion und die Einbeziehung vieler Interessengruppen.
Empfehlung **offen** **/** keine Empfehlung	„kann erwogen werden”	0	Zurzeit kann eine Empfehlung für oder gegen diese Intervention aufgrund bestimmter Gegebenheiten nicht getroffen werden (zum Beispiel unklares oder ausgeglichenes Nutzen‐Risiko‐Verhältnis, keine verfügbare Evidenz, et cetera)
***Schwache*** Empfehlung gegen eine Vorgehensweise	„kann nicht empfohlen werden“	↓	Wir sind der Auffassung, dass die meisten informierten Menschen, ein substanzieller Anteil jedoch nicht, eine Entscheidung gegen diese Intervention treffen würden.
***Starke*** Empfehlung gegen eine Vorgehensweise	„wird nicht empfohlen“	↓↓	Wir sind der Auffassung, dass alle oder fast alle informierten Menschen eine Entscheidung gegen diese Intervention treffen würden. In den meisten klinischen Situationen kann die Empfehlung als allgemeine Vorgehensweise übernommen werden.

Jede konsentierte Empfehlung wird in der Leitlinie von einer Box eingerahmt: die linke Spalte beinhaltet den Inhalt der Empfehlung unter Verwendung der standardisierten Begriffe beziehungsweise Leitliniensprache; die mittlere Spalte zeigt die Richtung und Stärke der Empfehlung und die rechte Spalte zeigt die Stärke des Konsenses in der Expertengruppe und die Evidenzbasis (Expertenkonsens versus evidenzbasiert).

*Für die Abschnitte „Konsensusprozess“, „Externer Review“, „Freigabe durch die Fachgesellschaften“, „Implementierung“, „Aktualisierung“, „Gültigkeit“ sowie „Netzwerk‐Metaanalysen: lesen und verstehen“ – siehe Langfassung*.

## ALLGEMEINE EMPFEHLUNGEN

### Einleitung und Auswahl einer systemischen Therapie

**Table:** 

ix‐1 | geprüft [2025] *Es wird empfohlen*, Wirksamkeit (siehe Abbildung 1 und Abbildung 2 sowie Zusammenfassung des Cochrane Reviews), Sicherheit (siehe Medikamentenkapitel), Komorbidität (siehe Tabelle 1 und Tabelle 2 in Publikation Teil 2 sowie jeweilige Kapitel in der Langfassung) und individuelle Patientenfaktoren bei der Auswahl einer systemischen Therapie bei mittelschwerer bis schwerer Psoriasis vulgaris zu berücksichtigen.	**↑↑**	starker konsens ^*^ Evidenz‐ und Konsensbasiert (siehe Evidenzbericht Kapitel 1) CoE: siehe Abbildung 2 und Abbildung 3
ix‐2 | modifiziert [2025] Bei Patienten mit mittelschwerer bis schwerer Psoriasis vulgaris *wird* die Einleitung einer systemischen Therapie entsprechend Abbildung 3 *empfohlen*.	**↑↑**	starker konsens ^*^ Evidenz‐ und Konsensbasiert (siehe Evidenzbericht Kapitel 1) CoE: siehe Abbildung 2 und Abbildung 3
ix‐3 | neu [2025] Bei der erstmaligen Einleitung einer Systemtherapie der Psoriasis *kann* insbesondere bei besonderer Schwere (siehe Kapitel „Definition des Schweregrades“) die Behandlung mit einem Biologikum mit einer Erstlinienzulassung (first‐line label) beziehungsweise mit Deucravacitinib *empfohlen werden*.	**↑**	starker konsens ^*^ Evidenz‐ und Konsensbasiert (siehe Evidenzbericht Kapitel 1) CoE: siehe Abbildung 2 und Abbildung 3

* Enthaltungen aufgrund von Interessenkonflikten: 5.

**ABBILDUNG 1:**
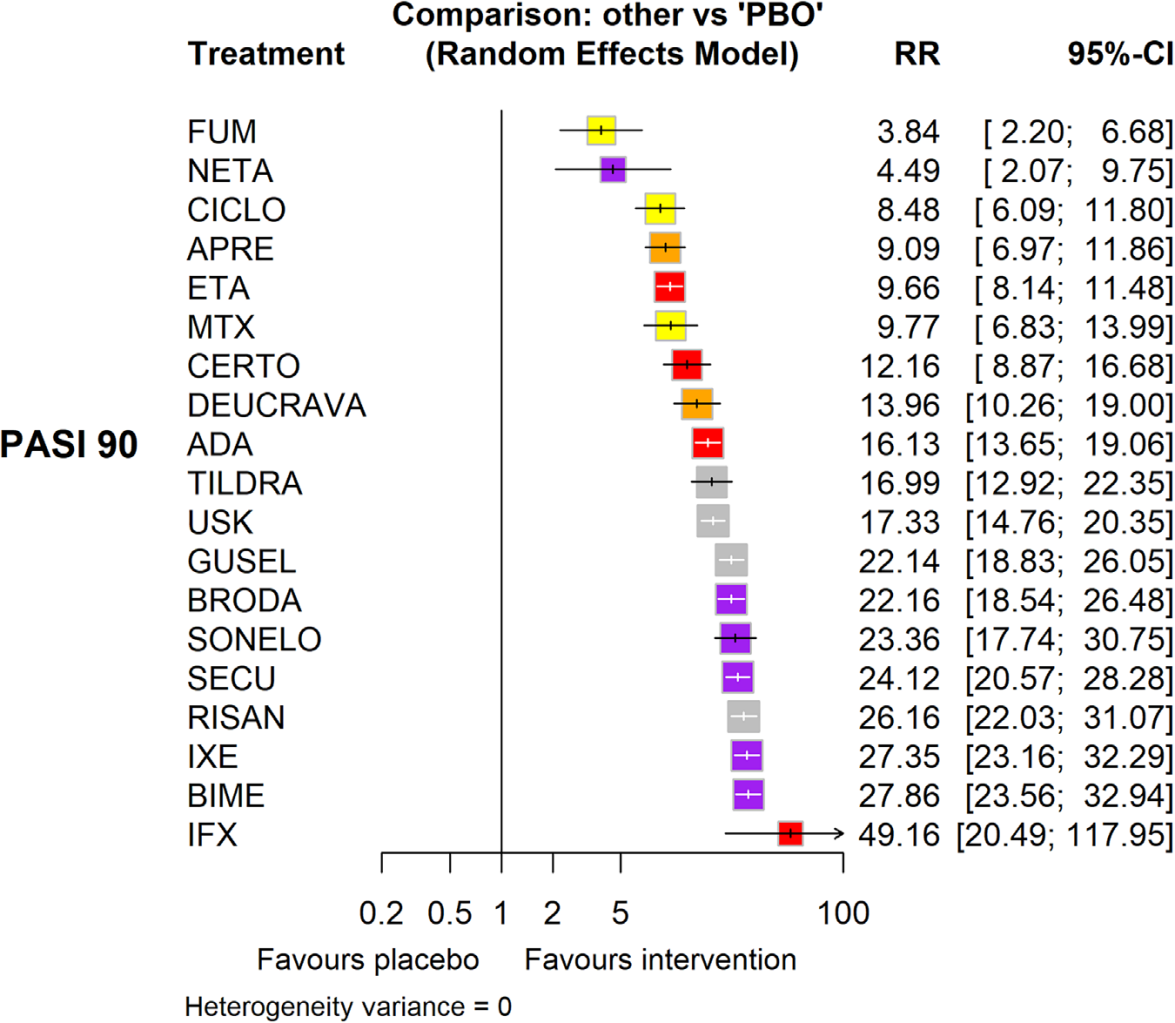
Forest‐Plot (relative Effekte versus Placebo), reproduziert nach [Copyright © 2023 The Cochrane Collaboration].^11^

**ABBILDUNG 2:**
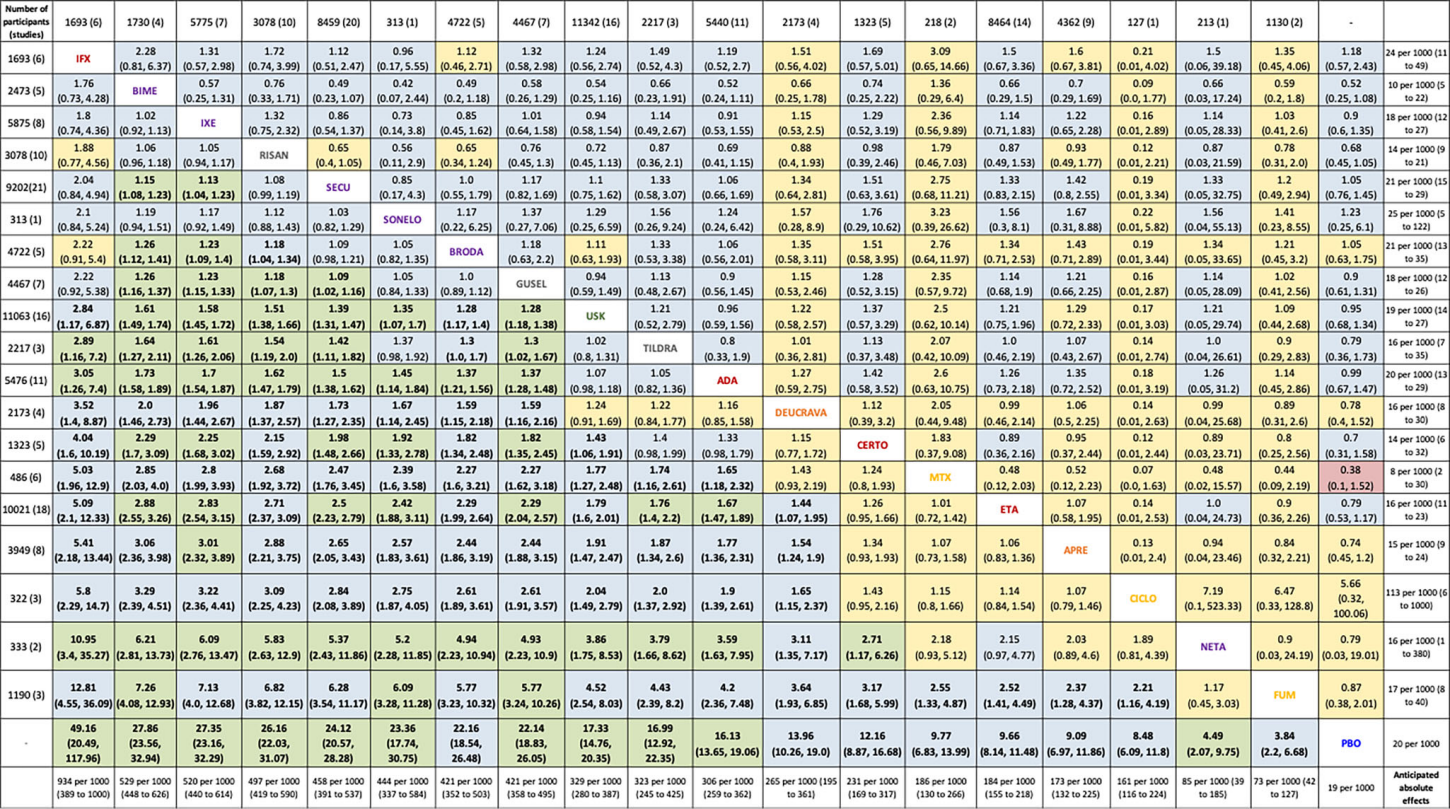
League‐Table, relativer Effekt (PASI 90: unteres Dreieck; SAE: oberes Dreieck), Certainty of Evidence (CINeMA): hoch (grün), moderat (blau), gering (gelb), sehr gering (rot). [Copyright © 2023 The Cochrane Collaboration].^11^

**TABELLE 2:** Empfohlene Laborkontrollen MTX.

Diagnostik	Zeitpunkt			
	*Vor der Behandlung*	*Innerhalb der ersten 2 Wochen*	*Innerhalb der ersten 3 Monate risikoadaptiert, zum Beispiel bei Diabetes mellitus, Adipositas/ Fettleber*	*Danach alle 3 Monate*
Blutbild (inklusive Differenzialblutbild)^*^	x	x	x	x
ALT, AST, Gamma‐GT, AP^**^	x		x	x
Serumkreatinin	x		x	x
Urinstatus	x			
Schwangerschaftstest (Urin oder Blut)	x			
Hepatitis‐B‐ und Hepatitis‐C‐Serologie	x			
HIV‐Serologie	x			
Serumalbumin^***^	x		x	x

Möglicherweise sind nicht alle Tests für alle Patienten notwendig. Anamnese, Risikoexposition und Patientencharakteristika müssen berücksichtigt werden. Je nach klinischen Anzeichen, Risiko und Exposition können weitere spezifische Tests erforderlich sein.

* Bei Leukozyten <3,0, Neutrophilen <1,0, Thrombozyten <100, Dosisreduktion oder Therapieende

** Bei Leberwerten > 2–3 x im Vergleich zum Ausgangswert: weitere Diagnostik einleiten (Bestimmung wiederholen/Vorstellung bei einem entsprechenden Facharzt) und Dosisreduktion oder Therapieende erwägen.

*** Bei ausgewählten Fällen (zum Beispiel bei Verdacht auf Hypoalbuminämie oder bei Patienten, die andere Medikamente mit hoher Bindungsaffinität für Serumalbumin einnehmen)

Die Empfehlungen zu Laborkontrollen basieren auf klinischer Erfahrung.

modifiziert [2025]| starker konsens; Enthaltungen aufgrund von Interessenkonflikten: 5

**ABBILDUNG 3:**
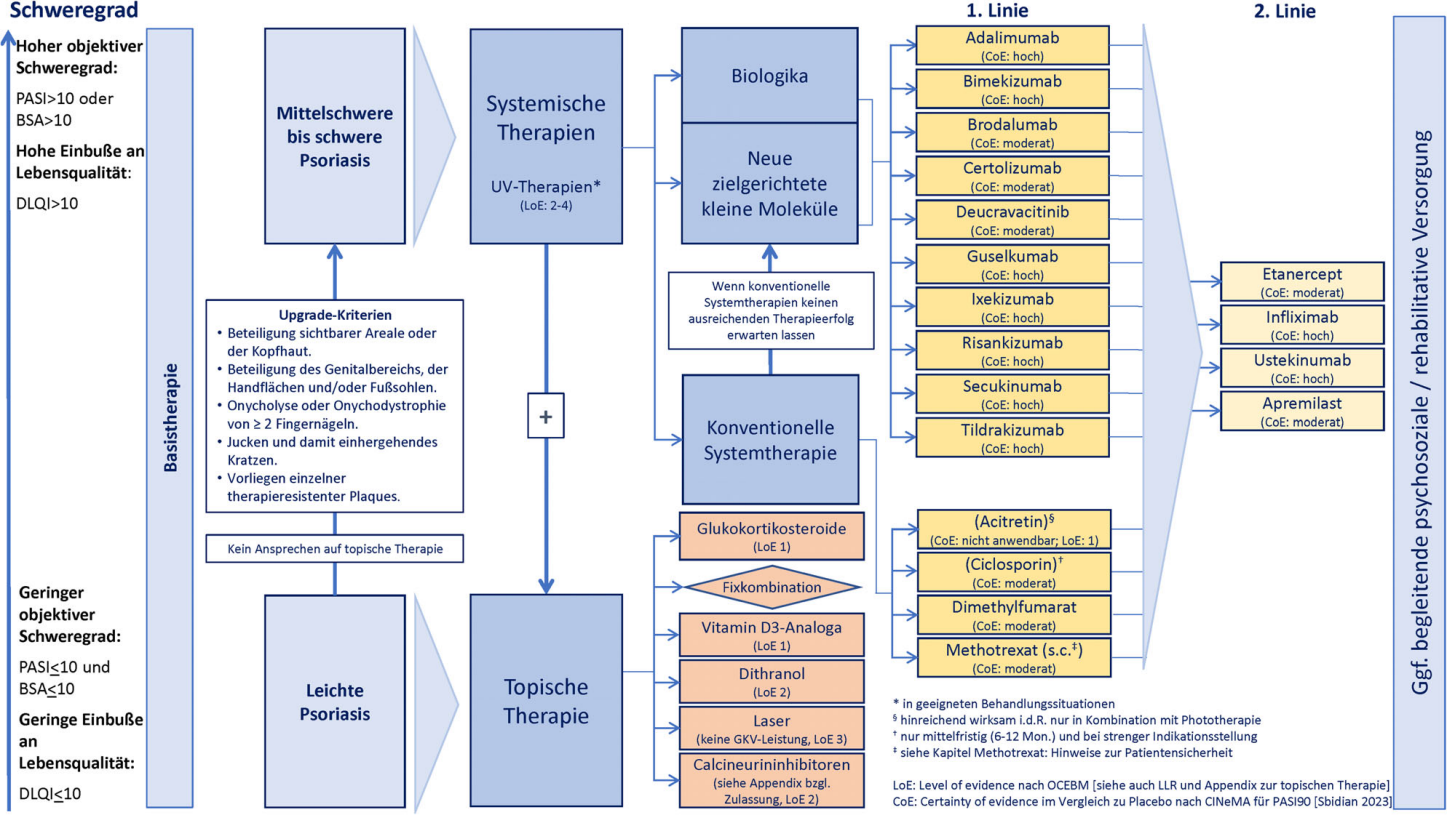
Übersicht der Therapieoptionen (CoE: siehe Abbildung 2 für Netzwerkvergleiche für PASI 90 sowie schwere unerwünschte Ereignisse).

#### Evidenzgrundlage

Die in Abbildung 3 aufgeführten systemischen Therapieoptionen und die Empfehlungen IX‐1 bis IX‐3 beruhen auf den Ergebnissen des Cochrane Reviews von Sbidian et al.^11^ Da dieser eine hohe methodische Qualität in der Bewertung mit dem AMSTAR‐II‐Instrument aufwies und in regelmäßigen Abständen aktualisiert wird, wurde auf eine Recherche nach weiteren systematischen Übersichtsarbeiten zu dieser Fragestellung verzichtet.

Die Aussagen zu topischen sowie UV‐Therapien beruhen auf der zuletzt im Jahre 2015 aktualisierten systematischen Evidenzsynthese, deren Ergebnisse im Appendix zur Leitlinie veröffentlicht wurden.

#### Begründung für die Abbildung 3 und die dazugehörigen Empfehlungen IX‐1 bis IX‐3

Die Abbildung 3 beschreibt die Behandlungsoptionen bei leichter sowie bei mittelschwerer bis schwerer Psoriasis. Der Übergang wird durch international konsentierte Upgrade‐Kriterien,^6^ definiert (siehe auch siehe Kapitel ii „Definition des Schweregrades“).

Da für den Endpunkt PASI 90 alle im Cochrane‐Review^11^ untersuchten Systemtherapien einen signifikanten Vorteil im Vergleich zu Placebo zeigten („*Certainty of evidence*“: hoch – moderat) und das relative Risiko für SAE sich nicht statistisch signifikant zwischen den jeweiligen Interventionen und Placebo unterschied, spricht die Leitliniengruppe die starke Positivempfehlung (IX‐2) für die Einleitung einer Systemtherapie bei Patienten mit mittelschwerer bis schwerer Psoriasis aus.

Die Ergebnisse der Netzwerkvergleiche für die Endpunkte PASI 90 und SAE allein ermöglichen es der Leitliniengruppe nicht, die Empfehlungsgrade für die einzelnen Wirkstoffe ausreichend zu differenzieren.

Mittlerweile stehen eine Vielzahl von Behandlungsmöglichkeiten zur Verfügung und die Kriterien für die Auswahl eines geeigneten Wirkstoffes sind komplex. Die Leitliniengruppe sieht es daher als notwendig an, neben Aspekten der Wirtschaftlichkeit auch die individuellen Patientenwünsche und ‐charakteristika, wie zum Beispiel Komorbiditäten oder Infektionsrisiken, zu evaluieren und diese zu konkreten Sicherheitsaspekten der einzelnen Wirkstoffe in Beziehung zu setzen (siehe auch Empfehlung IX‐1).

Einzelne Empfehlungsstärken ergeben sich somit individuell bei Betrachten der jeweiligen Patienten unter Berücksichtigung der Faktoren Wirksamkeit, Sicherheit, Komorbidität sowie weiterer individueller Patientenfaktoren.

Zur Unterstützung der Beurteilung der Eignung bei Komorbidität verweist die Leitliniengruppe neben der narrativen Evidenzdarstellung im folgenden Abschnitt auch auf die Wirkstoffkapitel (siehe Langfassung der Leitlinie), in denen besonders erwähnenswerte Aspekte der Fachinformationen abgebildet sind.

Darüber hinaus wurden in den Kapiteln zu besonderen klinischen Situationen und Komorbidität (siehe Langfassung der Leitlinie) Empfehlungen erarbeitet. In Tabelle 1 und Tabelle 2 in Publikation Teil 2 wurden diese Empfehlungen visualisiert und können Anwender der Leitlinie in der Entscheidungsfindung unterstützen.

Die Empfehlung IX‐3 zum direkten Einsatz der Erstlinien‐Biologika beziehungsweise Deucravacitinib begründet sich auf der besonders hohen Krankheitslast bei besonderer Schwere der Psoriasis und aufbauend auf den Ergebnissen des Cochrane‐Reviews^11^ (siehe auch Abbildung 2).

Die in Abbildung 3 vorgenommene Einteilung der Wirkstoffe in 1. Linie sowie 2. Linie erfolgte auf Basis der in den Fachinformationen aufgeführten zugelassenen Indikationen.

#### Ergebnisse der Netzwerk‐Metaanalyse^11^

“[…] Die Netzwerk‐Metaanalyse^11^ ergab, dass auf der Wirkstoffebene alle Interventionsgruppen (Nicht‐Biologika‐Systemtherapie, zielgerichtete kleine Moleküle, Biologika) einen höheren Anteil von Patienten aufwiesen, die den PASI 90 erreichten, als die Placebogruppe.“^11^ (Abbildung 1, 2).

„Bei der Behandlung mit IL‐17‐Inhibitoren war der Anteil der Patienten, die den PASI 90 erreichten höher als in allen anderen Interventionsgruppen.

Bei Behandlung mit IL‐17‐Inhibitoren, IL‐12/23‐Inhibitoren, IL‐23‐Inhibitoren sowie TNF‐Inhibitoren war der Anteil der Patienten, die den PASI 90 erreichten, höher als bei Behandlung mit Nicht‐Biologika‐Systemtherapie.

Für das Erreichen des PASI 90, waren im Vergleich zu Placebo die effektivsten Wirkstoffe (SUCRA Reihenfolge, für alle hohes Vertrauen in den Effektschätzer): Infliximab (relatives Risiko [RR] 49,16, 95%‐Konfidenzintervall [KI] 20.49–117,95), Bimekizumab (RR 27,86, 95%‐KI 23,56–32,94), Ixekizumab (RR 27,35, 95%‐KI 23,15–32,29), Risankizumab (RR 26,16, 95%‐KI 22,03–31,07).

Die klinische Wirksamkeit war im Vergleich der Wirkstoffe untereinander ähnlich. Bimekizumab und Ixekizumab erreichten mit signifikant höherer Wahrscheinlichkeit den PASI 90 als Secukinumab.

Bimekizumab, Ixekizumab und Risankizumab erreichten mit signifikant höherer Wahrscheinlichkeit den PASI 90 als Brodalumab und Guselkumab. Infliximab, IL‐17‐Inhibitoren (Bimekizumab, Ixekizumab, Secukinumab und Brodalumab) sowie IL‐23‐Inhibitoren (außer Tildrakizumab) erreichten mit signifikant höherer Wahrscheinlichkeit den PASI 90 als Deucravacitinib, Ustekinumab sowie die drei TNF‐Inhibitoren. Ustekinumab war gegenüber Certolizumab überlegen. Adalimumab, Tildrakizumab und Ustekinumab waren gegenüber Etanercept überlegen. Keine signifikanten Unterschiede zeigten sich zwischen Apremilast und Ciclosporin sowie MTX.“^11^ (Abbildung 2).

„Für das Risiko schwerer unerwünschter Ereignisse wurden keine signifikanten Unterschiede zwischen den Interventionen und Placebo gefunden. Das Risiko für schwere unerwünschte Ereignisse war in der Gruppe der mit MTX behandelten Patienten signifikant niedriger als bei den meisten anderen Interventionen.

Gleichwohl basierten die Analysen für schwere unerwünschte Ereignisse in allen Vergleichen auf einer sehr geringen Anzahl von Ereignissen (Spanne des Vertrauens in die Effektschätzer: sehr gering bis moderat). Die Ergebnisse müssen daher mit Vorsicht interpretiert werden.

Für andere Wirksamkeitsendpunkte (PASI75, PGA 0/1 waren die Ergebnisse ähnlich wie beim PASI 90. Ergebnisse zur Lebensqualität waren häufig nicht ausreichend berichtet beziehungsweise für viele Interventionen nicht vorhanden […]”.^11^

Methodisch ist anzumerken, dass die Endpunkte für den Zeitraum von 8 bis 24 Wochen nach Randomisierung evaluiert wurden, dass die Hauptanalyse – für die auch die *Certainty of Evidence* (CoE) bewertet wurde – auch Dosierungen beinhaltet, die nicht von der Europäischen Arzneimittel‐Agentur (EMA) zugelassen waren und dass bei den schweren unerwünschten Arzneimittelwirkungen (SAE) keine klare Trennung zwischen „therapiebedingten“ und „nicht therapiebedingten“ SAE erfolgte.

#### Weitere Aspekte zur Sicherheit

Neben den in der Netzwerk‐Metaanalyse berichteten Ergebnisse zu schweren unerwünschten Ereignissen hebt die Leitliniengruppe in den einzelnen Wirkstoffkapiteln erwähnenswerte Sicherheitsaspekte hervor.

#### Weitere Aspekte zum Wirkeintritt

Die für Patienten akzeptable Dauer bis zum Wirkeintritt ist ein individueller Faktor. Eine längere Zeit bis zum Wirkeintritt kann je nach Symptomatik der Patienten in Kauf genommen werden, wenn die Wirksamkeit des Wirkstoffes letztlich zufriedenstellend ist.

Aktuell wird die Möglichkeit der Modifikation des Krankheitsverlaufs der Psoriasis durch eine frühe systemische Therapieeinleitung durch verschiedene Studienansätze untersucht.^12^ Die Leitliniengruppe weist auf die laufende Diskussion hin und sieht hier in Zukunft möglichen Aktualisierungsbedarf für die Leitlinie, gegebenenfalls mit Einfluss auf Indikationsstellung und Therapieauswahl einer Systemtherapie.

*Für die Abschnitte „Empfehlungen zu Basistherapie“, „Topischer Therapie“, „Phototherapie“ sowie „Sonstige Therapien“ siehe Dokument Appendix A*.

## KONVENTIONELLE SYSTEMTHERAPIE

*Für die Abschnitte „Acitretin“, „Ciclosporin“ sowie das vollständige Kapitel „Dimethylfumarat“ siehe Langfassung*

### Dimethylfumarat

Zur Behandlung der Psoriasis ist in Europa ein DMF‐haltiges Medikament (Skilarence^®^) zugelassen und verfügbar. Die Herstellung des Fumarsäureesters Fumaderm^®^ wurde in Deutschland laut Information vom 18.12.2024 eingestellt.^13^

### Methotrexat (MTX)

Zum Vergleich der Wirksamkeit der oralen versus subkutanen MTX‐Verabreichung bei immunmediierten Erkrankungen liegen zwei Metaanalysen vor.^14^, ^15^ Eine der Metaanalysen aus dem Jahr 2019^14^ berichtete von einer höheren Wirksamkeit bei subkutaner Verabreichung, während die andere aus dem Jahr 2022^15^ keinen signifikanten Unterschied feststellte.

In Bezug auf die Toxizität zeigen einige Studien, dass die subkutane Verabreichung besser verträglich ist als die orale Verabreichung, während andere dies nicht gezeigt haben.^16^

Pharmakokinetische Studien deuten auf eine höhere Plasmabioverfügbarkeit und eine erhöhte Anreicherung von MTX‐Polyglutamaten (MTX‐PG) in roten Blutkörperchen (RBC) bei subkutaner Verabreichung während der initialen Behandlungsphase hin. Nach mehreren Monaten werden jedoch ähnliche intrazelluläre Wirkstoffspiegel bei beiden Verabreichungswegen beobachtet.^17^ Bei Dosierungen von über 15 mg wurde eine bessere Bioverfügbarkeit für die subkutane Gabe berichtet.^18^

Insbesondere wenn Dosierungen von über 15 mg gegeben werden oder das Risiko einer Überdosierung vermieden werden soll, ist die subkutane Zufuhr zu bevorzugen (da bei oraler Einnahme ein höheres Risiko einer Überdosierung besteht, weil die Patienten dazu neigen, die Tabletten mit größerer Wahrscheinlichkeit täglich anstatt einmal wöchentlich einnehmen). Die Leitliniengruppe verweist auf das Vorhandensein eines Rote‐Hand‐Briefes, der auf das Risiko der Überdosierung durch tägliche Einnahme explizit hinweist.^19^ Die empfohlene Anfangs‐ und Erhaltungsdosis beträgt in der Regel 15 mg MTX einmal wöchentlich. Bei unzureichendem Ansprechen kann die Dosis auf bis zu 20 mg MTX einmal wöchentlich erhöht werden. Eine weitere Erhöhung auf bis zu 25 mg MTX ist nur für eine kleine Untergruppe von Patienten von Vorteil. Eine darüberhinausgehende Erhöhung wird nicht empfohlen.^20^

#### Anwendungshinweise MTX

**Table:** 

*Vor der Behandlung*: Anamnese und klinische Untersuchung Objektive Erfassung des Schweregrades der Psoriasis (zum Beispiel durch PASI/BSA/PGA; Arthritis) Bestimmung der gesundheitsbezogenen Lebensqualität (zum Beispiel durch DLQI/Skindex‐29 oder ‐17) Laborkontrollen (siehe Tabelle 2) Ausschluss von schweren Nierenfunktionsstörungen (siehe auch Kapitel 3.6 Nierenerkrankung in Langfassung) Sichere Kontrazeption bei Frauen im gebärfähigen Alter (siehe auch Kapitel 3.11 Kinderwunsch/Schwangerschaft in Langfassung) Zur Notwendigkeit eines Konzeptionsschutzes bei männlichen Anwendern von MTX wird auf das Kapitel Kinderwunsch/Schwangerschaft sowie auf die jeweils gültigen Fachinformationen verwiesen. Bei auffälligen Leberwerten Überweisung des Patienten an einen entsprechende/n Facharzt zur Abklärung Prüfen des Impfstatus (siehe Kapitel Impfungen) *Während der Behandlung*: Anamnese und klinische Untersuchung Objektive Erfassung des Schweregrades der Psoriasis (zum Beispiel durch PASI/BSA/PGA; Arthritis) Bestimmung der gesundheitsbezogenen Lebensqualität (zum Beispiel durch DLQI/Skindex‐29 oder ‐17) Überprüfung der Begleitmedikation Laborkontrollen (siehe Tabelle 2) Effektive Kontrazeption bei Frauen im gebärfähigen Alter (siehe auch Kapitel 3.11 Kinderwunsch/ Schwangerschaft in der Langfassung) Zur Notwendigkeit eines Konzeptionsschutzes bei männlichen Anwendern von MTX wird auf das Kapitel Kinderwunsch/Schwangerschaft sowie auf die jeweils gültigen Fachinformationen verwiesen. 5 mg Folsäure einmal wöchentlich 24 Stunden nach der MTX‐Gabe Alkoholabstinenz empfehlen Bei Atemwegssymptomen (zum Beispiel bei Verdacht auf eine Alveolitis) Überweisung des Patienten an einen entsprechenden Facharzt zur Abklärung *Nach der Behandlung*: Zur Notwendigkeit eines Konzeptionsschutzes bei weiblichen beziehungsweise männlichen Anwendern über die Zeit der MTX‐Einnahme hinaus wird auf das Kapitel Kinderwunsch/Schwangerschaft in der Langfassung der Leitlinie sowie auf die jeweils gültigen Fachinformationen verwiesen.

modifiziert [2025] | starker konsens; Enthaltungen aufgrund von Interessenkonflikten: 5 | Nachabstimmung zur Nierenfunktionsstörung: starker konsens; Enthaltungen aufgrund von Interessenkonflikten: 6

#### Empfehlungen für Laborkontrollen

Siehe Tabelle 2

#### Unerwünschte Arzneimittelwirkungen

Für weitere Informationen/vollständige Darstellung siehe auch Fachinformationen und weitere Quellen. Die Leitliniengruppe beschloss, zu folgenden Aspekten Stellung zu nehmen:

Die wichtigsten unerwünschten Arzneimittelwirkungen (UAW) im Zusammenhang mit einer MTX‐Therapie sind die Mukositis, MTX‐induzierte Pneumonitis, Hepatotoxizität und die Myelosuppression.

Eine MTX‐induzierte Pneumonitis kann eine seltene, jedoch potenziell lebensbedrohliche Überempfindlichkeitsreaktion darstellen. Daher sind Patienten eingehend über die notwendige sofortige Kontaktaufnahme bei neu auftretendem trockenem Husten mit Dyspnoe ohne obere respiratorische Infektzeichen (Rhinitis, Sinusitis, Bronchitis) aufzuklären.

Die Frage, nach dem Monitoring bezüglich der möglichen Entstehung einer Leberfibrose ist immer wieder Bestandteil intensiver Diskussionen.^21^ Eine mögliche Überbewertung der Rolle von MTX für die Entstehung einer Leberfibrose wurde in verschiedenen Publikationen diskutiert und es wurde auf die Bedeutung der häufigen Komorbidität der Psoriasis mit Adipositas und Diabetes mellitus als bedeutenderer Faktor hingewiesen.^22^, ^23^, ^24^, ^25^

Die Bestimmung der Transaminasen ist das in der Leitliniengruppe übliches Vorgehen zum Monitorieren einer möglichen Leberschädigung durch MTX. Bei Auffälligkeiten soll ein entsprechender Facharzt konsultiert werden. Methoden zur weiteren Abklärung sind unter anderem die Berechnung des Fibrose (FIB)‐4‐Index sowie die Elastographie.^26^

Zusätzliche Risikofaktoren für eine Hepatotoxizität sind unter anderem Alkoholkonsum, Adipositas (BMI ≥ 30) und der nicht optimal eingestellte Diabetes mellitus.^27^, ^28^ Diese drei Risikofaktoren können zur Steatosis hepatis mit Steatohepatitis führen und somit auch die MTX‐Hepatotoxizität erhöhen.

Eine weitere seltene, jedoch gravierende Nebenwirkung bei der MTX‐Therapie ist die Myelosuppression mit einer geschätzten Häufigkeit von <1% (7/100 000 Patientenjahre).^29^ Diese Nebenwirkung resultiert in der Regel aus einer Überdosierung, zum Beispiel durch tägliche Applikation oder durch eine nicht beachtete beziehungsweise neu aufgetretene höhergradige Niereninsuffizienz bei einer Kreatinin‐Clearance von < 60 ml/min beziehungsweise < 30 ml/min. Die Aufklärung der Patienten über die frühen Symptome einer Panzytopenie (Halsschmerzen, Fieber, Stomatitis/Mundschleimhautbeschwerden und Blutungen) kann zur Früherkennung beitragen.

##### Auswahl wichtiger unerwünschter Arzneimittelwirkungen

**Table:** 

Sehr häufig	Übelkeit, Unwohlsein, Haarausfall
Häufig	Erhöhte Transaminasen, Myelosuppression, gastrointestinale Geschwüre
Gelegentlich	Fieber, Schüttelfrost, Depressionen, Infektionen
Selten	Nephrotoxizität, Leberfibrose und Leberzirrhose
Sehr selten	Eine interstitielle Lungenentzündung und Alveolitis

#### Besondere Aspekte während der Behandlung

Für weitere Informationen/vollständige Darstellung siehe auch Fachinformationen und weitere Quellen. Die Leitliniengruppe beschloss, zu folgenden Aspekten Stellung zu nehmen:

Bei Magen‐Darm‐Beschwerden während der MTX‐Therapie kann der Konsum von Kaffee und/oder dunkler Schokolade bei bis zu 30% der Patienten hilfreich sein.^30^

*Ältere Patienten*: Besondere Vorsicht ist bei der Behandlung geriatrischer Patienten geboten, bei denen die Dosierungen in der Regel niedriger als normalerweise sein sollten. Darüber hinaus sollte bei ihnen die Nierenfunktion regelmäßig überwacht werden. (CAVE bei Kreatinin‐Clearance <60 ml/min, insbesondere bei zusätzlich bestehender diuretischer Therapie, ACE‐Hemmer Medikation, warme Wetterlage).

#### Wichtige Kontraindikationen

Für weitere Informationen/vollständige Darstellung siehe auch Fachinformationen und weitere Quellen. Die Leitliniengruppe beschloss, zu folgenden Aspekten Stellung zu nehmen:

*Absolute Kontraindikationen*

- Schwere Infektionen
- Schwere Lebererkrankungen
- Schwere Nierenfunktionsstörungen
- Aktiver Kinderwunsch von Frauen im gebärfähigen Alter, Schwangerschaft/Stillzeit (siehe Kapitel „Schwangerschaft“)
- Alkoholmissbrauch
- Knochenmark‐Insuffizienz/hämatologische Veränderungen
- Akutes Magengeschwür
- Deutlich reduzierte Lungenfunktion
- Mangelndes Verständnis über die Applikation einmal pro Woche

*Relative Kontraindikationen*

- Nieren‐ oder Lebererkrankungen
- Hohes Alter
- Colitis ulcerosa
- Gastritis
- Fettleibigkeit (BMI > 30)
- Diabetes mellitus (entgleist, Kreatinin‐Clearance < 60 ml/min und Albuminurie > 30 mg/g, Kreatinin im Spontanurin)^31^, ^32^
- Maligne Erkrankungen (siehe auch Kapitel „Krebs“)

#### Arzneimittelinteraktionen

Für weitere Informationen/vollständige Darstellung siehe auch Fachinformationen und weitere Quellen. Die Leitliniengruppe beschloss, zu folgenden Aspekten Stellung zu nehmen:

Eine Reihe von Medikamenten, einschließlich Salicylate, Sulfonamide, Diphenylhydantoin und einige Antibiotika (das heißt Penicillin, Tetracycline, Chloramphenicol, Trimethoprim), können die Bindung von MTX an Serumalbumin vermindern und damit das Risiko einer MTX‐Toxizität erhöhen. Die tubuläre Sekretion wird durch Probenecid gehemmt. Besondere Vorsicht ist bei Patienten geboten, die gleichzeitig Azathioprin oder Retinoide verwenden. Einige NSAR können den MTX‐Spiegel und damit auch die MTX‐Toxizität erhöhen. Daher wird empfohlen, NSAR zu anderen Tageszeiten als MTX zu verabreichen. Folsäure sollte im Abstand zur MTX‐Applikation 24 Stunden versetzt eingenommen werden. Da MTX nach 24 Stunden im Blut nicht mehr nachweisbar ist, wird durch die Folsäuregabe keine Wirkungsabschwächung erwartet. Es gibt einige Hinweise darauf, dass die Kombination von MTX und Folsäure unerwünschte Reaktionen verringern kann, ohne dabei die Wirksamkeit zu beeinträchtigen.^33^, ^34^, ^35^

##### Aufstellung der wichtigsten Arzneimittelwechselwirkungen

**Table:** 

**Substanzen**	**Art der Wechselwirkung**
Colchicin, Ciclosporin (CsA), NSAID, Penicillin, Probenecid, Salicylate, Sulfonamide	Verringerte renale Elimination von MTX
Chloramphenicol, Cotrimoxazol, Zytostatika, Ethanol, NSAID, Pyrimethamin, Sulfonamide	Erhöhtes Risiko für Knochenmark‐ und Magen‐Darm‐Toxizität
Barbiturate, Cotrimoxazol, Phenytoin, Probenecid, NSAID, Sulfonamide	Wechselwirkung mit der Plasmaproteinbindung
Ethanol, Leflunomid, Retinoide, Tetracycline	Erhöhte Hepatotoxizität

*Maßnahmen im Falle einer Überdosierung: siehe Langfassung*.

## THERAPIE MIT BIOLOGIKA UND NEUEN ZIELGERICHTETEN KLEINEN MOLEKÜLEN

*Für die Abschnitte „Adalimumab“, „Apremilast“, „Brodalumab“, „Certolizumab Pegol“, „Etanercept“, „Guselkumab“, „Infliximab“, „Ixekizumab“, „Risankizumab“, „Secukinumab“, „Tildrakizumab“, „Ustekinumab“ sowie „neu zugelassene Medikamente und Therapien in der Entwicklung“ siehe Langfassung*.

### Bimekizumab

#### Anwendungshinweise Bimekizumab

**Table:** 

*Vor der Behandlung*: Anamnese und körperliche Untersuchung einschließlich bisher erhaltener Therapien, Malignome, Infektionen (unter anderem Candidainfektionen) und entzündlicher Darmerkrankungen. Meldung des/der Patient*in an ein Psoriasisregister prüfen Objektive Erfassung des Schweregrades der Psoriasis (wie zum Beispiel durch PASI/BSA/PGA; Arthritis) Bestimmung der gesundheitsbezogenen Lebensqualität (zum Beispiel durch DLQI/Skindex‐29 oder ‐17) Zu den empfohlenen Maßnahmen gehören außerdem: Ausschluss Malignome der Haut Untersuchung auf Lymphadenopathie Laborkontrollen (siehe Tabelle 3) Ausschluss einer Tuberkulose (siehe Kapitel Tuberkulose) Ausschluss aktiver Infektionen Prüfen des Impfstatus Sichere Kontrazeption *Während der Behandlung*: Anamnese und körperliche Untersuchung mit Fokus auf Infekte (insb. der oberen Atemwege, Candida, Tuberkulose), Kontrazeption und Symptome einer entzündlichen Darmerkrankung Objektive Erfassung des Schweregrades der Psoriasis (zum Beispiel durch PASI/BSA/PGA; Arthritis) Bestimmung der gesundheitsbezogenen Lebensqualität (zum Beispiel durch DLQI/Skindex‐29 oder ‐17) Laborkontrollen (siehe Tabelle 3) *Nach der Behandlung*: Nach Absetzen einer Therapie mit Bimekizumab: Nachverfolgung mit Anamnese und körperlicher Untersuchung. Für Informationen über die Notwendigkeit der Fortsetzung der Verhütung unmittelbar nach Absetzen der Therapie mit einem Biologikum siehe Kapitel “Kinderwunsch/Schwangerschaft”

neu [2025] | konsens, Enthaltungen aufgrund von Interessenkonflikten: 4

**TABELLE 3:** Empfohlene Laborkontrollen Bimekizumab.

Diagnostik	Zeitpunkt	
	*Vor der Behandlung*	*Alle 3–6 Monate*
Blutbild (inklusive Differenzialblutbild)	x	x
Leberwerte	x	x
Schwangerschaftstest (Urin oder Blut)	x	
Hepatitis‐B‐ und Hepatitis‐C‐Serologie	x	
HIV‐Serologie	x	
Interferon‐Gamma‐Release‐Assay (IGRA) (Ausschluss Tuberkulose)	x	

Möglicherweise sind nicht alle Tests für alle Patienten notwendig. Anamnese, Risikoexposition und Patientencharakteristika müssen berücksichtigt werden. Je nach klinischen Anzeichen, Risiko und Exposition können weitere spezifische Tests erforderlich sein.

Die Empfehlungen zu Laborkontrollen basieren auf klinischer Erfahrung.

neu [2025]| konsens; Enthaltungen aufgrund von Interessenkonflikten: 5

#### Empfehlungen für Laborkontrollen

Siehe Tabelle 3

#### Unerwünschte Arzneimittelwirkungen

Für weitere Informationen und eine vollständige Auflistung wird auch auf Fachinformationen und weitere Quellen verwiesen. Die Leitliniengruppe hat beschlossen, zu folgenden Aspekten Stellung zu nehmen:

Aus Sicht der Leitliniengruppe weist Bimekizumab, mit Ausnahme von Candidosen, ein ähnliches Sicherheitsprofil auf wie andere IL‐17‐Antagonisten wie Ixekizumab und Secukinumab sowie der IL‐17R‐Antagonist Brodalumab.

In allen Phase‐III‐Studien (BE READY^36^, BE VIVID^37^, BE SURE^38^ und BE RADIANT^39^) wurde Bimekizumab gut vertragen.

Charakteristika der gepoolten Analyse von Gordon et al.^40^: Es wurde ein Pooling von Sicherheitsdaten aus einer Kohorte mit Patienten aus vier randomisierten klinischen Phase‐II‐Studien (BE ABLE 1, BE ABLE 2, PS0016 und PS0018) und vier randomisierten klinischen Phase‐III‐Studien (BE VIVID, BE READY, BE SURE und BE BRIGHT) durchgeführt.^40^ In dieser Analyse wurden insgesamt 1789 Patienten (1252 [70,0%] Männer; Durchschnittsalter [SD], 45,2 [13,5] Jahre) mit einer oder mehreren Dosen von Bimekizumab behandelt. Die gesamte Exposition gegenüber Bimekizumab betrug 3109,7 Personenjahre.^40^ Behandlungsbedingte unerwünschte Ereignisse (TEAE) traten mit einer expositionsadjustierten Inzidenzrate (EAIR) von 202,4 pro 100 Personenjahre auf und nahmen mit zunehmender Bimekizumab‐Exposition nicht zu.^40^ EAIR für suizidale Gedanken und Verhaltensweisen (0,0 pro 100 Personenjahre; 95%‐KI 0,0–0,2 pro 100 Personenjahre) sowie schwerwiegende kardiovaskuläre Ereignisse (0,5 pro 100 Personenjahre; 95%‐KI 0,3–0,8 pro 100 Personenjahre) waren gering.^40^

*Neutropenie*: Die EAIR für Neutropenie betrug (0,8 pro 100 Personenjahre; 95%‐KI 0,6–1,2 pro 100 Personenjahre).^40^

*Infektionen*: Die drei am häufigsten berichteten TEAE waren Nasopharyngitis (19,1 pro 100 Personenjahre; 95%‐KI 17,4–20,9 pro 100 Personenjahre), orale Candidiasis (12,6 pro 100 Personenjahre; 95%‐KI 11,3–14,0 pro 100 Personenjahre) und Infektionen der oberen Atemwege (8,9 pro 100 Personenjahre; 95%‐KI 7,8–10,1 pro 100 Personenjahre).^40^ Die meisten Fälle von oraler Candidiasis waren leicht oder mäßig stark ausgeprägt; drei Ereignisse führten zu einem Abbruch der Behandlung.^40^

*Entzündliche Darmerkrankung*: Zu Patienten mit entzündlichen Darmerkrankungen (CED) liegen nur begrenzt Daten vor. In der oben beschriebenen gepoolten Analyse von Gordon et al.^40^ waren die EAIR für entzündliche Darmerkrankungen (0,1 pro 100 Personenjahre; 95%‐KI 0,0–0,3 pro 100 Personenjahre) gering. Bei bekannter Vorgeschichte von Morbus Crohn wurden Patienten von den klinischen Phase‐III‐Studien ausgeschlossen. Ein Fall von Colitis ulcerosa wurde unter Bimekizumab gemeldet. Es wird zur Vorsicht bei der Verschreibung von Bimekizumab bei Patienten mit einer Vorgeschichte von CED geraten.

*Candidiasis*: Neben den unter der Zwischenüberschrift „Infektionen“ berichteten Ergebnissen der gepoolten Analyse von Gordon et al.^40^ wurden die Ergebnisse einer Phase‐III‐Studie^39^ herangezogen. In der Studie BE RADIANT^39^ wurde bei erwachsenen Patienten mit moderater bis schwerer Psoriasis Bimekizumab mit Secukinumab verglichen. Die Häufigkeit von Candidainfektionen über den Zeitraum von 0 bis 48 Wochen war bei den mit Bimekizumab behandelten Personen (n = 79/373 [21,2%]) höher als bei den mit Secukinumab behandelten (n = 17/370 [4,6%]).^39^

Die duale Hemmung von IL‐17A und IL‐17F könnte die Schutzfunktion der Schleimhäute stärker beeinträchtigen und somit das Risiko einer oralen Candidiasis erhöhen. Eine frühzeitige Behandlung von Candida‐Infektionen mit topischer oder systemischer Therapie wird empfohlen. Für weitere Informationen zur Behandlung von Candidiasis wird auf Fachinformationen von Antimykotika oder auf (internationale) Leitlinien verwiesen.^41^, ^42^, ^43^. Im Falle wiederkehrender mykotischer Infektionen kann ein Wechsel der Psoriasis‐Therapie erwogen werden. Jedoch ist zu beachten, dass klinisch signifikante, schwere Infektionen immer eine Kontraindikation für alle Biologika darstellen.

#### Besondere Aspekte während der Behandlung

Für weitere Informationen und eine vollständige Auflistung wird auch auf Fachinformationen und weitere Quellen verwiesen. Die Leitliniengruppe hat beschlossen, zu folgenden Aspekten Stellung zu nehmen:

*Chirurgie*: Zu Operation bei Patienten, die mit Bimekizumab behandelt werden, sind der Leitliniengruppe aktuell keine Daten bekannt. Die Entscheidung, Bimekizumab vor einer Operation abzusetzen, sollte auf individuellen Faktoren beruhen, wie zum Beispiel Art und Risiko des chirurgischen Eingriffs, Patientencharakteristika, Schweregrad der Psoriasis bei eventuellem Abbruch der Behandlung und so weiter Eine Rücksprache mit dem behandelnden Chirurgen wird empfohlen.

#### Wichtige Kontraindikationen

Für weitere Informationen und eine vollständige Auflistung wird auch auf Fachinformationen und weitere Quellen verwiesen. Die Leitliniengruppe hat beschlossen, zu folgenden Aspekten Stellung zu nehmen:

*Absolute Kontraindikationen*:

- Klinisch relevante aktive Infektionen

*Relative Kontraindikationen*:

- Schwangerschaft oder Stillzeit
- Chronisch entzündliche Darmerkrankungen

*Für den Abschnitt „Arzneimittelinteraktionen und Maßnahmen im Falle einer Überdosierung“ siehe Langfassung*.

### Deucravacitinib

#### Anwendungshinweise Deucravacitinib

**Table:** 

*Vor der Behandlung*: Anamnese und körperliche Untersuchung (insbesondere bereits erhaltene Therapien, Malignome sowie Infektionszeichen und ‐risiken) Meldung des Patienten an ein Psoriasisregister prüfen Objektive Erfassung des Schweregrades der Psoriasis (wie zum Beispiel durch PASI/BSA/PGA; Arthritis) Bestimmung der gesundheitsbezogenen Lebensqualität (zum Beispiel durch DLQI/Skindex‐29 oder ‐17) Zu den empfohlenen Maßnahmen gehören außerdem: Ausschluss Malignome der Haut Untersuchung auf Lymphadenopathie Ausschluss einer Tuberkulose (siehe Kapitel Tuberkulose) Ausschluss aktiver und chronischer Infektionen Prüfen des Impfstatus entsprechend aktueller Impfempfehlungen inklusive prophylaktische Impfung gegen Herpes Zoster Laborkontrollen (siehe Tabelle 4) Ausschluss von Schwangerschaft oder Stillen Verlässliche Kontrazeption Hinweis an Patienten, die Behandlung abzubrechen und sich zur Untersuchung vorzustellen, wenn Muskelschwäche oder ‐schmerzen verspürt werden, insbesondere wenn diese von Abgeschlagenheit oder Fieber begleitet werden *Während der Behandlung*: Anamnese und klinische Untersuchung, inklusive Erhebung von Risikofaktoren für schwere Infektionen, Infektionszeichen und Malignome Objektive Erfassung des Schweregrades der Psoriasis (wie zum Beispiel durch PASI/BSA/PGA; Arthritis) Bestimmung der gesundheitsbezogenen Lebensqualität (zum Beispiel durch DLQI/Skindex‐29 oder ‐17) Laborkontrollen (siehe Tabelle 4) Verlässliche Kontrazeption

**Table:** 

*Nach der Behandlung*: Nach Absetzen einer Therapie mit Deucravacitinib Nachverfolgung mit Anamnese und körperlicher Untersuchung Für Informationen über die Notwendigkeit der Fortsetzung der Kontrazeption unmittelbar nach Absetzen der Therapie siehe Kapitel “Kinderwunsch/Schwangerschaft”

neu [2025] | starker konsens; Enthaltungen aufgrund von Interessenkonflikten: 5.

**TABELLE 4:** Empfehlungen für Laborkontrollen Deucravacitinib.

Diagnostik	Zeitpunkt	
	*Vor der Behandlung*	*Nur bei entsprechenden anamnestischen oder klinischen Hinweisen*
Blutbild (inklusive Differenzialblutbild)	x	(x)
Leberwerte	x	(x)
Serumkreatinin	x	(x)
Schwangerschaftstest (Urin oder Blut)	x	(x)
Hepatitis‐B‐ und Hepatitis‐C‐Serologie	x	(x)
HIV‐Serologie	x	(x)
Creatinkinase (CK)	x	(Bei Muskelschmerzen während der Behandlung)
Interferon‐Gamma‐Release‐Assay (IGRA) (Ausschluss Tuberkulose)	x	(x)

Möglicherweise sind nicht alle Tests für alle Patienten notwendig. Anamnese, Risikoexposition und Patientencharakteristika müssen berücksichtigt werden. Je nach klinischen Anzeichen, Risiko und Exposition können weitere spezifische Tests erforderlich sein.

Die Empfehlungen basieren auf Expertenmeinungen und berücksichtigen, dass die Erfahrungen mit dem Arzneimittel noch begrenzt sind. Die empfohlenen Laborkontrollen gehen über die in der Fachinformation^44^ (Stand: Juli 2024) vorgeschlagenen Laborkontrollen hinaus.

neu [2025] | konsens, Enthaltungen aufgrund von Interessenkonflikten: 6.

#### Empfehlungen für Laborkontrollen

Siehe Tabelle 4

#### Unerwünschte Arzneimittelwirkungen

Für weitere Informationen/vollständige Darstellung siehe auch Fachinformation^44^ und weitere Quellen.^45^, ^46^, ^47^, ^48^ Die Leitliniengruppe beschloss, zu folgenden Aspekten Stellung zu nehmen:

Die häufigsten unerwünschten Arzneimittelwirkungen (die in ≥ 1% und mit einer höheren Rate als in der Placebo‐Gruppe auftraten) in den kombinierten Daten aus den POETYK‐PSO‐1‐ und POETYK‐PSO‐2‐Studien bis zur Woche 16 waren obere Atemwegsinfektionen, erhöhte Creatinkinase‐Werte im Blut, Herpes simplex, Mundgeschwüre, Follikulitis und Akne. Kopfschmerzen, Durchfall und Übelkeit wurden ebenfalls berichtet, und zwar in ähnlicher Häufigkeit in der Deucravacitinib‐Gruppe wie in der Placebo‐Gruppe. Bis zur Woche 52 wurden keine neuen unerwünschten Arzneimittelwirkungen identifiziert, und ihre Inzidenzraten stiegen nicht im Vergleich zu denen, die in den ersten 16 Wochen der Behandlung beobachtet wurden.

##### Übersicht wichtiger Nebenwirkungen (Deucravacitinib)

**Table:** 

Sehr häufig	Infekte der oberen Atemwege^*^
Häufig	Herpes simplex Infektionen^**^, Orale Ulzerationen^***^, akneiforme Ausschläge^****^, Follikulitis
Gelegentlich	Herpes zoster

* Nasopharyngitis, obere Atemwegsinfektion, virale obere Atemwegsinfektion, Pharyngitis, Sinusitis, akute Sinusitis, Rhinitis, Tonsillitis, Peritonsillarabszess, Laryngitis, Tracheitis und Rhinotracheitis

** Lippenherpes, Herpes simplex, Genitalherpes und andere Herpesvirusinfektionen

*** Aphthen, Ulzerationen der Mundschleimhaut, Zungenulzera und Stomatitis

**** Akne, akneiforme Dermatitis, Exanthem, Rosazea, Pusteln, papulopustulöse Hautveränderungen

##### Infektionen

Deucravacitinib kann das Infektionsrisiko erhöhen. Die Mehrheit der beobachteten Infektionen war nicht schwerwiegend, sondern leicht bis mittelschwer; darunter Infektionen der oberen Atemwege, die nicht zum Abbruch der Behandlung führten. Die häufigsten schwerwiegenden Infektionen, die unter Deucravacitinib berichtet wurden, waren Lungenentzündungen und COVID‐19, was auf die pandemische Situation zurückgeführt werden kann.

Reaktivierungen von Herpesviren (zum Beispiel Herpes zoster, Herpes simplex) wurden in klinischen Studien gemeldet. Die meisten Fälle von Herpes zoster waren leicht bis mittelschwer, auf ein Dermatom beschränkt, verliefen günstig und führten nicht zum Absetzen der Behandlung. Während der Zulassungsstudien POETYK PSO‐1, PSO‐2 und der Open‐Label‐Verlängerungsstudie waren zehn von 18 Patienten, die von Herpes zoster berichteten, jünger als 50 Jahre, und es gab einen Fall von Herpes zoster mit Beteiligung mehrerer Dermatome bei einem immunkompetenten Probanden, der Deucravacitinib erhielt. Ärzte sollten die Patienten über frühe Anzeichen und Symptome von Herpes zoster aufklären und darauf hinweisen, dass eine Behandlung so früh wie möglich erfolgen sollte.

##### Veränderung von Laborwerten

Gepoolte Daten aus klinischen Studien zu veränderten Laborwerten zeigen, dass es unter Behandlung mit Deucravacitinib zu folgenden Laborveränderungen kommen kann: Anstieg der Creatinkinase (von asymptomatisch bis hin zur Rhabdomyolyse), Anstieg der Triglyceridwerte und Leberenzym‐Erhöhungen um das ≥ 3‐fache des oberen Normalwertes. Die Deucravacitinib‐Behandlung soll unterbrochen werden, wenn eine Myopathie oder Leberschädigung vermutet wird. Die Patienten sollten angewiesen werden, sich zur Untersuchung vorzustellen, wenn sie Muskelschwäche oder ‐schmerzen verspüren, insbesondere wenn diese von Abgeschlagenheit oder Fieber begleitet werden.

##### Malignome

In den gepoolten Daten aus den gesamten Behandlungszeiträumen der Zulassungsstudien PSO‐1, PSO‐2 und der offenen Verlängerungsstudie (insgesamt 2482 Patientenjahre unter Exposition mit Deucravacitinib) wurden Malignitäten bei 22 Patienten gemeldet (0,9 pro 100 Patientenjahre), darunter elf Fälle von nichtmelanozytärem Hautkrebs (0,4 pro 100 Patientenjahre) und drei Fälle von Lymphomen (0,1 pro 100 Patientenjahre).

#### Besondere Aspekte während der Behandlung

Für weitere Informationen/vollständige Darstellung siehe auch Fachinformation^44^ und weitere Quellen.^47^, ^48^, ^49^, ^50^ Die Leitliniengruppe beschloss, zu folgenden Aspekten Stellung zu nehmen:

##### Potenzielle Risiken in Zusammenhang mit JAK‐Inhibition

Sicherheitsbedenken führten dazu, dass die *U.S. Food and Drug Administration* (FDA) und die EMA Maßnahmen befürworteten, um das Risiko schwerwiegender kardiovaskulärer Ereignisse, Malignome, thrombotischer Ereignisse und Tod im Zusammenhang mit Januskinase (JAK)‐Inhibitoren zu minimieren.

Es ist nicht bekannt, ob Deucravacitinib mit den beobachteten oder potenziellen unerwünschten Reaktionen anderer JAK‐Inhibitoren in Verbindung gebracht werden kann. Deucravacitinib ist ein hochselektiver TYK2‐Inhibitor mit minimaler oder keiner Aktivität gegen JAK 1/2/3 bei klinisch relevanten Dosen und Konzentrationen. Der allosterische Mechanismus der TYK2‐Inhibition verringert das Risiko von *Off‐Target*‐Effekten, und Daten aus PSO‐1, PSO‐2 und der nicht verblindeten Verlängerungsstudie zeigten konsistente Sicherheitsprofile von Deucravacitinib bei Patienten mit Psoriasis. Trotzdem sind weitere Beobachtungen erforderlich, um die Langzeitsicherheit von Deucravacitinib vollständig zu charakterisieren.

##### Chirurgie

Es liegen keine Daten zum Management von Operationen bei Patienten vor, die mit Deucravacitinib behandelt werden. Die Entscheidung zur Unterbrechung der Deucravacitinib‐Therapie vor einer Operation sollte von Fall zu Fall getroffen werden. Die Art und das Risiko des operativen Eingriffs, die Patientencharakteristika, das Risiko einer Infektion sowie das Risiko einer Verschlechterung der Psoriasis sollten berücksichtigt werden. Es wird empfohlen, sich mit dem Chirurgen zu beraten.

Die Leitlinie des *American College of Rheumatology/American Association of Hip and Knee Surgeons* für das perioperative Management von antirheumatischen Medikamenten bei Patienten mit rheumatischen Erkrankungen, die sich einer elektiven Totalhüft‐ oder Kniearthroplastik unterziehen, empfiehlt, die JAK‐Inhibitoren mindestens 3 Tage vor der Operation abzusetzen.

#### Wichtige Kontraindikationen

Für weitere Informationen/vollständige Darstellung siehe auch Fachinformation^44^ und weitere Quellen.^45^, ^46^, ^47^ Die Leitliniengruppe beschloss, zu folgenden Aspekten Stellung zu nehmen:

*Absolute Kontraindikationen*

- Überempfindlichkeit gegenüber dem Wirkstoff oder einem der Hilfsstoffe
- Aktive Tuberkulose oder andere aktive schwere Infektionen

*Relative Kontraindikationen*

- Schwere Lebererkrankung
- Schwangerschaft

Die Risiken und Vorteile der Behandlung mit Deucravacitinib sollten sorgfältig abgewogen werden, bevor die Therapie bei Patienten begonnen wird, die chronische oder wiederkehrende Infektionen haben, die ein Risiko für eine Tuberkulose haben, die schwere oder opportunistische Infektionen in der Anamnese aufweisen oder die Grunderkrankungen haben, die sie anfällig für Infektionen machen können.

#### Arzneimittelinteraktionen

Für weitere Informationen/vollständige Darstellung siehe auch Fachinformation^44^ und weitere Quellen.^47^, ^51^ Die Leitliniengruppe beschloss, zu folgenden Aspekten Stellung zu nehmen:

Ergebnisse aus Studien an gesunden Probanden zeigten, dass keine klinisch signifikanten Unterschiede in der Pharmakokinetik von Deucravacitinib beobachtet wurden, wenn es zusammen mit Arzneimitteln verabreicht wurde, die verschiedene Arzneimittel verstoffwechselnde Enzyme und Transporter hemmen oder induzieren. Unter anderem: Ciclosporin (duales Pgp/BCRP‐Inhibitor), Fluvoxamin (CYP1A2‐Inhibitor), Ritonavir (CYP1A2‐Induktor), Diflunisal (UGT1A9‐Inhibitor), Pyrimethamin (OCT1‐Inhibitor), Famotidin (H2‐Rezeptor‐Antagonist) oder Rabeprazol (Protonenpumpenhemmer). Es wurden keine klinisch signifikanten Unterschiede in der Pharmakokinetik der folgenden Medikamente beobachtet, wenn sie zusammen mit Deucravacitinib verabreicht wurden: Rosuvastatin, Methotrexat, Mycophenolatmofetil und orale Kontrazeptiva (Norethindronacetat und Ethinylestradiol).

Die Kombinationstherapie von Deucravacitinib mit anderen immunmodulatorischen Wirkstoffen, einschließlich Biologika oder Phototherapie, wurde bei Plaque‐Psoriasis nicht evaluiert.

#### Überdosierung/Maßnahmen im Fall einer Überdosierung

„Deucravacitinib wurde gesunden Probanden in Einzeldosen von bis 40 mg (> das 6‐Fache der für den Menschen empfohlenen Dosis von 6 mg/Tag) und in mehreren Dosen von bis zu 24 mg/Tag (12 mg zweimal täglich) über einen Zeitraum von 14 Tagen gegeben, ohne dass eine dosislimitierende Toxizität aufgetreten ist.

Im Falle einer Überdosis wird empfohlen, den Patienten auf Anzeichen und Symptome von Nebenwirkungen zu überwachen und unverzüglich eine geeignete symptomatische Behandlung einzuleiten. Durch eine Dialyse wird Deucravacitinib nicht in wesentlichem Umfang aus dem systemischen Kreislauf entfernt.“^44^

## BIOSIMILARS

Zum Zeitpunkt der Erstellung dieser Leitlinie waren in Europa Biosimilars für Adalimumab, Etanercept, Infliximab und Ustekinumab^52^ erhältlich. Die Empfehlungen dieser Leitlinie gelten jeweils gleichermaßen für das originale Molekül als auch für die entsprechenden Biosimilars.

*Für die Abschnitte „Hilfestellungen für besondere klinische Situationen und bei Vorliegen von Komorbiditäten“ siehe Teil 2 der Leitlinie im JDDG‐Folgeheft*.

## HINWEIS ZUR LEITLINIENADAPTATION

Beim Verfassen dieser Leitlinie haben die Autoren*innen auf einer Vorversion der folgenden Publikation aufgebaut und diese außerdem adaptiert, neu zusammengesetzt und übersetzt: EUROGUIDERM GUIDELINE ON THE SYSTEMIC TREATMENT OF PSORIASIS von Nast A et al., deren finale Fassung auf der Webseite des European Dermatology Forum (https://www.guidelines.edf.one/guidelines/psoriasis‐guideline) zur Verfügung steht (lizenziert unter CC BY NC 4.0, https://creativecommons.org/licenses/by‐nc/4.0/):

− A Nast, PI Spuls, C Dressler, Z Bata‐Csörgö, I Bogdanov, H Boonen, EMGJ De Jong, I Garcia‐Doval, P Gisondi, D Kaur‐Knudsen, S Mahil, T Mälkönen, JT Maul, S Mburu, L Mercieca, U Mrowietz, A Pennitz, E Remenyik, D Rigopoulos, PG Sator, M Schmitt‐Egenolf, M Sikora, K Strömer, O Sundnes, G Van Der Kraaij, N Yawalkar, C Zeyen, C Smith. EUROGUIDERM GUIDELINE FOR THE SYSTEMIC TREATMENT OF PSORIASIS VULGARIS September 2023, partial update February 2025.

Darüber hinaus basieren die Inhalte des hier vorliegenden Artikels auf einer Adaptation der Vorversionen der Leitlinie, die in ihrer finalen Form wie folgt publiziert wurde:

− Nast, A., Altenburg, A., Augustin, M., Boehncke, W.‐H., Härle, P., Klaus, J., Koza, J., Mrowietz, U., Ockenfels, H.‐M., Philipp, S., Reich, K., Rosenbach, T., Schlaeger, M., Schmid‐Ott, G., Sebastian, M., von Kiedrowski, R., Weberschock, T. and Dressler, C. (2021), Deutsche S3‐Leitlinie zur Therapie der Psoriasis vulgaris, adaptiert von EuroGuiDerm – Teil 1: Therapieziele und Therapieempfehlungen. JDDG: Journal der Deutschen Dermatologischen Gesellschaft, 19: 934‐951. https://doi.org/10.1111/ddg.14508_g
− Nast, A., Altenburg, A., Augustin, M., Boehncke, W.‐H., Härle, P., Klaus, J., Koza, J., Mrowietz, U., Ockenfels, H.‐M., Philipp, S., Reich, K., Rosenbach, T., Schlaeger, M., Schmid‐Ott, G., Sebastian, M., von Kiedrowski, R., Weberschock, T. and Dressler, C. (2021), Deutsche S3‐Leitlinie zur Therapie der Psoriasis vulgaris, adaptiert von EuroGuiDerm – Teil 2: Therapiemonitoring, besondere klinische Situationen und Komorbidität. JDDG: Journal der Deutschen Dermatologischen Gesellschaft, 19: 1092‐1117. https://doi.org/10.1111/ddg.14507_g

Die vorliegende adaptierte Leitlinie hat das Freigabeverfahren des European Dermatology Forum nicht durchlaufen, sondern wurde von den herausgebenden deutschen Fachgesellschaften freigeben. Diese Leitlinie unterliegt den Bestimmungen der Creative Commons Attribution‐NonCommercial.

## DANKSAGUNG

Open access Veröffentlichung ermöglicht und organisiert durch Projekt DEAL.

## INTERESSENKONFLIKT

Für die Autoren der deutschen Version siehe Leitlinienreport der deutschen Adaption auf https://register.awmf.org/de/leitlinien/detail/013‐001. Für die Autoren der EuroGuiDerm‐Version siehe Methods Report: https://www.guidelines.edf.one/guidelines/psoriasis‐guideline [Zuletzt aufgerufen 10. April 2025].
